# BRAF Mutations in Myeloid Neoplasms: Prevalence, Co-Mutation Landscape, and Clinical Outcomes—A Comprehensive Review

**DOI:** 10.3390/biomedicines14030672

**Published:** 2026-03-15

**Authors:** Shehab F. Mohamed, Ali Mohamed, Mohamed Fawzi Mudarres, Azza E. A. Abdalla, Abdulrahman F. Al-Mashdali, Mohammed Abdulgayoom, Rowan Mesilhy, Tareq Abuasab, Honar Cherif, Gautam Borthakur

**Affiliations:** 1 Department of Hematology, National Center for Cancer Care and Research (NCCCR), Hamad Medical Corporation, Doha 3050, Qatar; aalmashdali@hamad.qa (A.F.A.-M.); mmohammed35@hamad.qa (M.A.); hcherif@hamad.qa (H.C.); 2 Department of Internal Medicine, Rochester General Hospital, Rochester, NY 14621, USA; ali.s.mohamedmd@gmail.com (A.M.); azza.abdalla@rochesterregional.org (A.E.A.A.); 3 Department of Internal Medicine, Mercy Hospital, Saint Louis, MO 63141, USA; fawzimud@gmail.com; 4 Department of Internal Medicine, Hamad Medical Corporation, Doha 3050, Qatar; rmesilhy@hamad.qa; 5 Department of Internal Medicine, Section of Hematology, Baylor College of Medicine, Houston, TX 77030, USA; tareq.abuasab@bcm.edu; 6 College of Medicine, Qatar University, Doha 2713, Qatar; 7 Department of Leukemia, The University of Texas MD Anderson Cancer Center, Houston, TX 77030, USA; gborthak@mdanderson.org

**Keywords:** BRAF, myeloid neoplasms, acute myeloid leukemia, chronic myelomonocytic leukemia, MAPK pathway, therapy-related AML, molecular genetics, targeted therapy

## Abstract

**Background:** BRAF is a core component of the RAS–MAPK signaling pathway and an established oncogenic driver in several solid tumors and selected hematologic malignancies. In myeloid neoplasms, BRAF mutations are rare, and their prevalence, molecular context, and clinical significance remain incompletely defined. Available evidence is scattered across heterogeneous reports involving acute myeloid leukemia, myelodysplastic syndromes, myeloproliferative neoplasms, and overlap myelodysplastic/myeloproliferative neoplasms, with variable descriptions of mutation subtypes, co-mutational profiles, cytogenetic associations, therapeutic approaches, and clinical outcomes. To address these gaps, this review synthesizes data from the published literature up to 2025, summarizing the distribution, genetic landscape, and clinical impact of molecularly confirmed BRAF mutations across the spectrum of myeloid neoplasms. **Results:** Across published cohorts, BRAF mutations occurred in less than 1% of unselected myeloid neoplasms, with enrichment in chronic myelomonocytic leukemia and therapy-related or secondary acute myeloid leukemia. Both V600E and non-V600E variants were observed, typically within a complex genomic background involving ASXL1, TET2, DNMT3A, SRSF2, and RAS-pathway mutations. Acute myeloid leukemia cases showed poor prognosis, with median overall survival measured in months, whereas myelodysplastic syndromes and chronic myelomonocytic leukemia demonstrated relatively longer survival. Targeted MAPK inhibition produced hematologic responses in selected cases but rarely resulted in durable molecular clearance. **Conclusions:** BRAF mutations in myeloid neoplasms are rare, heterogeneous, and usually represent secondary events in clonal evolution. Although mutation clearance appears prognostically relevant, current targeted approaches provide limited durability, underscoring the need for prospective studies in this setting.

## 1. Introduction

**Figure 1:**
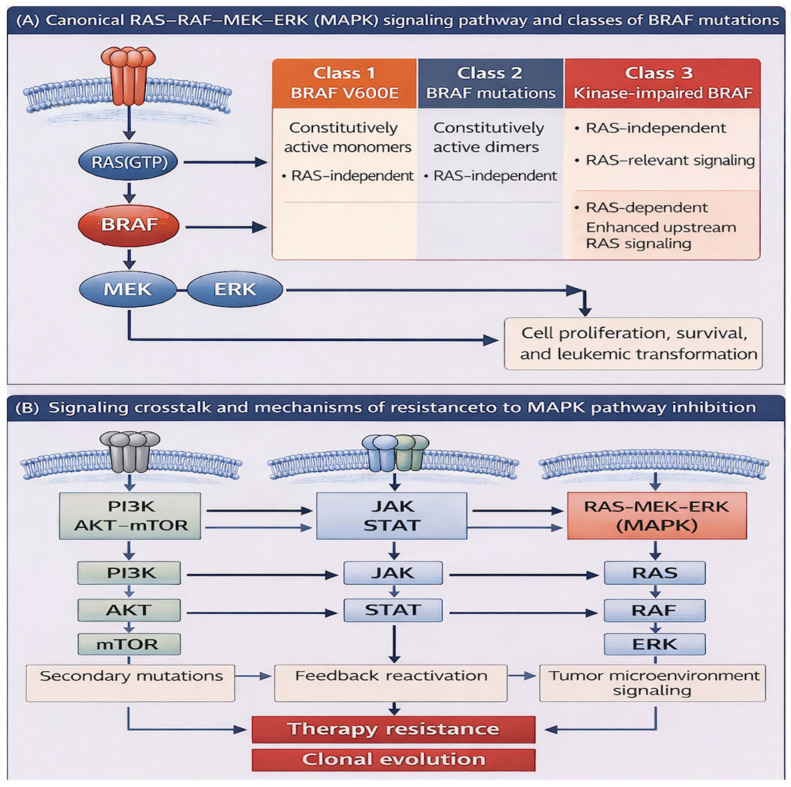
Overview of MAPK signaling and BRAF mutation classes in myeloid neoplasms. (**A**) The canonical RAS-RAF-MEK-ERK signaling cascade activated by receptor tyrosine kinases (RTKs). Activating BRAF mutations are classified into three functional classes: class 1 mutations (e.g., V600E), which signal as constitutively active monomers independent of RAS; class 2 mutations, which signal as constitutive dimers and are also RAS-independent; and class 3 mutations, which are kinase-impaired and enhance upstream RAS-dependent signaling. (**B**) Crosstalk between MAPK signaling and other major intracellular pathways relevant to leukemogenesis, including PI3K–AKT–mTOR and JAK–STAT signaling pathways.

The B-Raf proto-oncogene, serine/threonine kinase (BRAF) is a key component of the RAS–MAPK/ERK signaling pathway, which regulates cellular proliferation, differentiation, and survival [1]. Located on chromosome 7q34, BRAF encodes a 766–amino acid protein with three conserved regions: CR1, which contains the RAS-binding domain (RBD); CR2, a serine-rich hinge region; and CR3, the kinase domain responsible for MEK phosphorylation [2]. Under normal conditions, BRAF remains inactive through intramolecular interactions, with activation occurring when GTP-bound RAS engages the RBD. This conformational shift halts autoinhibition, promotes membrane localization and dimerization, and enables phosphorylation of downstream MEK–ERK targets [1,3,4]. The canonical RAS–RAF–MEK–ERK signaling cascade and its interaction with other major signaling pathways implicated in leukemogenesis are illustrated in Figure 1. Oncogenic mutations disrupt this regulation, driving constitutive pathway activation and uncontrolled proliferation [2].

BRAF mutations are established oncogenic drivers in multiple solid tumors, including melanoma (approximately 60%), papillary thyroid carcinoma (40–60%), colorectal cancer (10–15%), and non-small-cell lung cancer (1–4%), as well as subsets of glioblastoma and lymphoma [2,5,6]. More than 200 variants have been described, with nearly 30 functionally characterized. These mutations fall into three classes: class 1 (e.g., V600E), which act as constitutively active monomers; class 2, which signal as active dimers independent of RAS; and class 3, which are kinase-impaired but amplify upstream RAS activity [1,7]. This classification has therapeutic relevance, as class 1 and 2 tumors often respond to direct BRAF inhibition, whereas class 3 mutations are typically dependent on upstream RAS or receptor tyrosine kinase signaling [7].

In hematologic malignancies, BRAF mutations are rare. They are clinically significant in hairy cell leukemia, where V600E serves as both a diagnostic marker and therapeutic target [8,9], but have been reported in fewer than 1–2% of myeloid neoplasms. Notably, the spectrum of BRAF mutations in myeloid disease appears to include a higher proportion of non-V600E variants, whose functional and clinical significance remain unclear [5]. Given the scarcity of cases, available data is limited to small series and case reports with heterogeneous methods and outcomes. This review synthesizes published evidence up to 2025 on molecularly confirmed BRAF mutations across the major categories of myeloid neoplasms, including acute myeloid leukemia, myelodysplastic syndromes, myeloproliferative neoplasms, and myelodysplastic/myeloproliferative overlap disorders. We integrate available data on mutation prevalence, variant subtype, variant allele frequency, co-occurring genetic alterations, cytogenetic features, treatment strategies, and clinical outcomes, with particular attention to patterns of mutation acquisition, therapeutic response, and prognostic implications. By consolidating these dispersed observations, this review aims to clarify the emerging biological and clinical landscape of BRAF-mutated myeloid neoplasms and to highlight areas requiring further investigation.

## 2. Results

### 2.1. Study Characteristics

Across 12 published studies, research designs included retrospective and prospective cohorts, cross-sectional analyses, and case series. Sample sizes ranged from 2 patients to more than 6000. Mutation detection methods also varied over time.

Early studies used PCR-based assays, such as restriction fragment length polymorphism (RFLP) analysis [10], single-stranded conformational polymorphism (SSCP) [11,12], and allele-specific PCR for BRAF V600E [13].

In contrast, more recent studies relied on next-generation sequencing (NGS). These included targeted myeloid panels of different sizes, such as 28-gene, 53-gene, or 81-gene assays [14], as well as broader clinical panels like the HopeSeq Heme panel [15]. Other approaches included capture-based and amplicon-based sequencing [16,17,18,19,20].

### 2.2. Prevalence, Clinical Features, and Genetic Landscape

**Figure 2:**
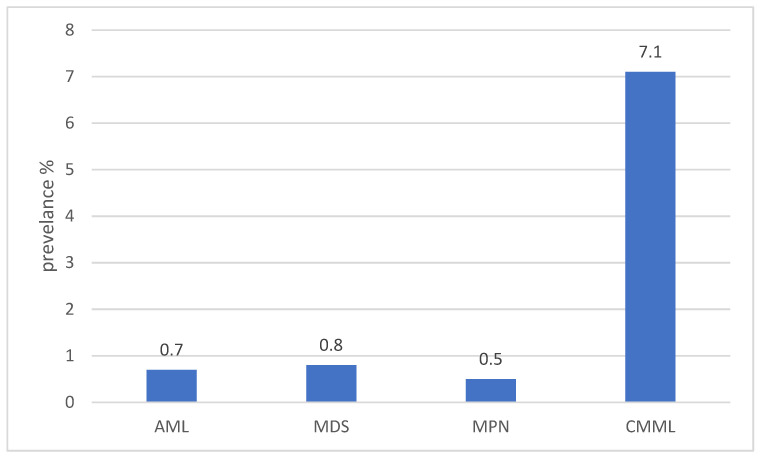
Distribution of BRAF mutations across myeloid neoplasms. Prevalence values reflect representative estimates reported in published cohorts ([14,15,16,20,21]). Data illustrate the relative enrichment of BRAF mutations in CMML compared to AML, MDS, and MPN, rather than precise pooled estimates.

The overall prevalence of BRAF mutations in myeloid neoplasms was consistently low, generally below 1%. Reported frequencies were 0.5–1% in acute myeloid leukemia (AML), myelodysplastic syndromes (MDS), and chronic myelomonocytic leukemia (CMML), though CMML-specific cohorts demonstrated higher rates, reaching up to 7.1% (Figure 2). Sporadic cases were also described in acute promyelocytic leukemia (APL), myeloid sarcoma, and biphenotypic acute leukemias (Table 1).

**Table 1:** Summary of published studies on BRAF mutations in myeloid neoplasms.

ID	Study	DOI	Study Design	Total Sample Size	BRAF Prevalence	Age	Gender (M/F)
1	Abuasab T et al. (2024) [14]	10.1080/10428194.2024.2347539	Retrospective cohort study	6667	48/6667 (0.7%) - AML: 18/2438 (0.7%) - MDS/CMML: 22/2538 (0.8%) - MPN: 5/939 (0.5%)	Median: 70 yo (24–89)	28/20
2	Zhang et al. (2014) [16]	10.1002/ajh.23652	Retrospective cohort study	70	5/70 (7.1%)	Median: 67.8 yo (28–86)	37/33
3	Christiansen et al. (2005) [10]	10.1038/sj.leu.2404009	Retrospective cohort study	140	t-AML: 3/51 (5.8%) t-MDS: 0	47, 65, 69 yo	1/2
4	Fei et al. (2024) [15]	10.3390/ijms25105183	Retrospective cohort study	2632	14/2632 (0.53%)	Mean: 63.9 yo (23–89)	10/4
5	George et al. (2024) [17]	10.3390/genes15111383	Case series	2	16/1600 (1%) 2 cases detailed	2, 75 yo	2/0
6	Abu-Shihab et al. (2023) [18]	10.1093/heqpro/daad094	Cross-sectional study	42	NA	Median: 67 yo (19–84)	24/18
7	Kandarpa et al. (2017) [19]	10.1002/ajh.24728	Retrospective cohort study	8	2/7	65, 83 yo	1/1
8	Santos et al. (2014) [21]	10.1182/blood.V124.2.1.3172.3172	Case series	87	1/87 (1.2%)	NA	NA
9	Papaemmanuil et al. (2016) [20]	10.1056/NEJMoa1516192	Prospective cohort study	1540	9/1540 (0.6%)	18–84 yo	823/719
10	Xu et al. (2017) [22]	10.1080/10428194.2016.1213830	Cross-sectional study	339	4/339 (1%)	0.5, 49, 59, 60 yo	2/2
11	Lee et al. (2004) [11]	10.1038/sj.leu.2403201	Retrospective cohort study	90	2/90 (2.2%)	20–80 yo	NA
12	Lee et al. (2025) [23]	10.1101/2025.10.14.682328	Retrospective cohort study	5779	50/5779 (1%)	Median: 67 yo (19–84)	27/23

Abbreviations: yo = years old; M = male; F = female; NA = not available; AML = acute myeloid leukemia; MDS = myelodysplastic syndrome; MPN = myeloproliferative neoplasm; CMML = chronic myelomonocytic leukemia; t-AML = therapy-related AML. Note: For studies reporting multiple myeloid neoplasm (MN) subtypes, the number of cases in each subtype is indicated within the corresponding table cell. Across larger cohorts, BRAF-mutated patients were typically older adults, with median ages in the sixth to seventh decade; sex distribution was generally balanced when reported [10,11,14,15,16,17,18,19,20,21,22]. Mutations occurred across a range of myeloid neoplasms (Table 2). However, they were relatively enriched in chronic myelomonocytic leukemia and in therapy-related or secondary AML, where frequencies exceeded those seen in de novo AML [11,14,18].

**Figure 3:**
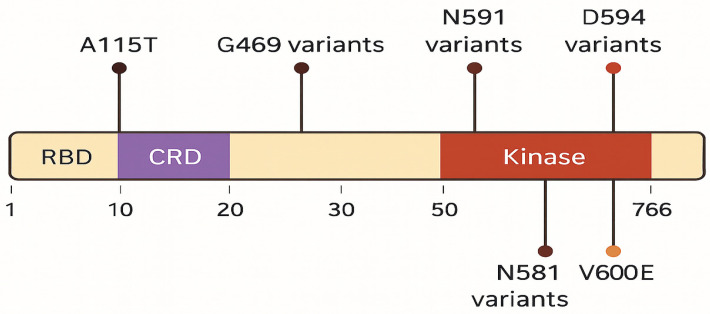
Distribution of BRAF mutations across functional protein domains.

**Figure 4:**
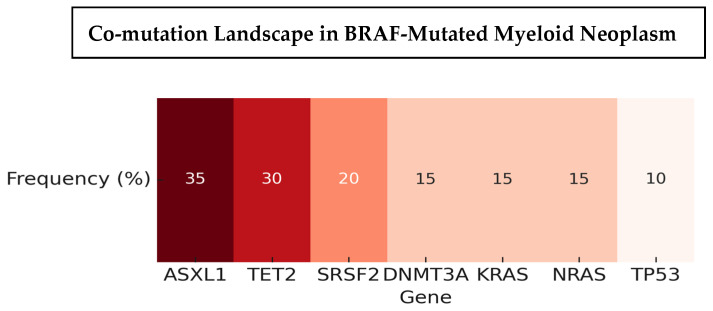
Heatmap showing recurrent co-mutations in BRAF-mutated myeloid neoplasms. Frequencies reflect aggregated estimates from the largest available studies ([14,15,18,20]). Values are illustrative to highlight recurrent mutation partners; exact frequencies vary by cohort and sequencing method.

The spectrum of BRAF variants was heterogeneous, including V600E, G469A/V, D594E/G, L597Q/R, N581S/I/K, and K601E, with variant allele frequencies ranging from 1% to over 80% (Figure 3). Co-mutations were frequent and recurrent across studies. The most common partners included ASXL1, TET2, DNMT3A, and splicing factor mutations such as SRSF2, along with recurrent RAS-pathway lesions (KRAS/NRAS) (Figure 4). Other reported partners included IDH1, EZH2, FLT3-ITD, RUNX1, JAK2, TP53, and NPM1. Only a minority of cases carried BRAF mutations as solitary events, suggesting that BRAF usually occurs within a multi-hit genomic context. Cytogenetic profiles ranged from normal karyotype to high-risk abnormalities, such as monosomy 7, complex karyotypes, and KMT2A rearrangements (Table 3).

**Table 2:** Underlying disease and classification of BRAF-mutated cases in published studies.

ID	Study	Total BRAF-Mutated Cases	Underlying Disease Category (n)	Reported Subtype/Clinical Classification (n)
1	Abuasab et al., 2024 [14]	48	AML (18), CMML (12), MDS (10), MF (5), MDS/MPN (1), APL (1), Myeloid sarcoma (1)	s-AML (12), t-MDS (4)
2	Zhang et al., 2014 [16]	5	CMML	CMML-1 (2), CMML-2 (3)
3	Christiansen et al. (2005) [10]	3	Therapy-related AML	AML-M5
4	Fei et al. (2024) [15]	14	AML (7), MPN (3), MDS (2), MDS/MPN (1), unclear (1)	Acute monocytic AML; AML with monocytic differentiation; ET
5	George et al. (2024) [17]	2	s-AML	AML with monocytic features
6	Abu-Shihab et al. (2023) [18]	32	AML	De novo AML (19), relapsed/refractory AML (8), secondary AML (15)
7	Kandarpa et al. (2017) [19]	2	MPN	Post-ET myelofibrosis
8	Santos et al. (2014) [21]	1	MDS/MPN overlap	Ph-negative MDS/MPN-U
9	Papaemmanuil et al. (2016) [20]	9	AML	De novo AML; therapy-related AML; secondary AML
10	Xu et al. (2017) [22]	4	AML	Monoblastic AML
11	Lee et al. (2004) [11]	2	AML	Biphenotypic AML (1); AML with maturation (1)
12	Lee et al. (2025) [23]	50	AML	De novo AML (21), secondary AML (20), relapsed/refractory AML (9), AML-MR (34)

Abbreviations: AML = acute myeloid leukemia; MDS = myelodysplastic syndrome; MPN = myeloproliferative neoplasm; CMML = chronic myelomonocytic leukemia; MF = myelofibrosis; APL = acute promyelocytic leukemia; ET = essential thrombocythemia; s-AML = secondary AML; MDS/MPN-U = myelodysplastic/myeloproliferative neoplasm, unclassifiable; AML-MR = Acute Myeloid Leukemia with Myelodysplasia-Related changes. Note: For studies reporting multiple myeloid neoplasm (MN) subtypes, the number of cases in each subtype is indicated within the corresponding table cell.

**Table 3:** Genetic characteristics of BRAF-mutated myeloid neoplasm cases.

ID	Study	BRAF Mutation/VAF	Co-Mutations	Karyotype/Cytogenetics
1	Abuasab et al., 2024 [14]	G469A, V600E, others; median VAF 8.6% (1.3–86.6)	KRAS, NRAS, ASXL1, TET2, SRSF2, TP53, CBL, DNMT3A	44% diploid; 19% high-risk; poor risk in AML
2	Zhang et al., 2014 [16]	D594E, N581S, L597Q, G466E (exon 11/15)	RAS WT	Low risk
3	Christiansen et al. (2005) [10]	V600E	AML1, CBFb, MLL, RARa, KRAS	Recurrent balanced translocations; +8; MLL-rearrangements
4	Fei et al. (2024) [15]	V600E, D594G, N581S, others (exons 6, 11, 15, 17)	NRAS, KRAS, DNMT3A, TET2, ASXL1, IDH1, JAK2, TP53, etc.	Normal: 6; Abnormal: 7 (del5q, +8, del9q, del20q, etc.); KMT2A fusions
5	George et al. (2024) [17]	V600E, N581S	TET2, KRAS, ZRSR2, EZH2, RUNX1T1, RAF1	Complex karyotype; KMT2A rearrangements
6	Abu-Shihab et al. (2023) [18]	G469, D594, others; VAF 1–83%	TET2, ASXL1, NRAS, KRAS, RUNX1, DNMT3A, FLT3, NPM1, SRSF2	NA
7	Kandarpa et al. (2017) [19]	D594E, V600E, G469V	JAK2, ASXL1, ASXL2, TP53, NF1, PIK3R3, KMT2C	NA
8	Santos et al. (2014) [21]	D594G	JAK2	del(5q)
9	Papaemmanuil et al. (2016) [20]	V600E, D594N, L597Q, A115T	ASXL1, DNMT3A, EZH2, FLT3, IDH1/2, NRAS, RUNX1, TP53, etc.	Favorable: 205; Intermediate: 960; Adverse: 253
10	Xu et al. (2017) [22]	V600E, D594G, K601E (exons 11,15)	NPM1, ASXL	Normal: 2; Abnormal: +8, der (1;12), t(10;11)
11	Lee et al. (2004) [11]	Exon 11	NA	NA
12	Lee et al. (2025) [23]	V600, G469, D594, others Median VAF 15% (1–83%)	TET2, NPM1, NRAS, KRAS, BRAF, TP53, and SRSF2.	MECOM-rearrangements, RUNX1:RUNX1T1 fusion

Abbreviations: VAF = variant allele frequency; NA = not available; WT = wild type. Cytogenetic notation follows ISCN standards.

### 2.3. Survival Outcomes

Survival outcomes varied by disease subtype and genetic background. In AML, prognosis was generally poor, with median overall survival (OS) ranging from 126 days to 7 months [14,15,17,18]. Clearance of the BRAF mutation, when documented, correlated with improved outcomes (OS 34.8 vs. 10.4 months, *p* = 0.047) [11,14]. In MDS and CMML, survival was somewhat longer, typically 16–22 months [11,14,17], particularly in patients with diploid cytogenetics and fewer co-mutations (Supplementary Table S2) In many cases, BRAF mutations occurred within complex genomic backgrounds that included established adverse-risk mutations (e.g., ASXL1, TP53, DNMT3A, and RAS-pathway genes), making it difficult to attribute clinical outcomes specifically to BRAF alterations.

### 2.4. Treatment Approaches and Outcomes

Differences in treatment strategies and clinical outcomes across disease subtypes are summarized in Table 4. In AML, most patients received intensive induction chemotherapy, with generally poor outcomes; occasional prolonged survival was observed in those who achieved BRAF clearance. MAPK inhibitors (dabrafenib, trametinib, vemurafenib) were used mainly in relapsed or refractory settings, producing transient hematologic or morphologic responses without durable molecular remissions [24,25,26,27,28,29]. In MDS and CMML, hypomethylating agents were the most common therapy, with modest clinical improvements but rare molecular clearance; hydroxyurea was also frequently used in CMML. In MPN, patients generally received conventional agents such as hydroxyurea or ruxolitinib, with no consistent evidence for BRAF-targeted therapy.

Outcomes differed substantially by disease subtype. AML harboring BRAF mutations carried the poorest prognosis, with median OS typically ranging from 4 to 7 months [14,17,19]. Clearance of the BRAF mutation, when achieved, was associated with longer survival (34.8 vs. 10.4 months) and may be enhanced by HSCT [11]. Secondary and therapy-related AML were enriched for high-risk cytogenetic abnormalities, including KMT2A rearrangements and monosomy 7, and were associated with inferior outcomes, often measured in weeks [10,15,21]. In contrast, de novo AML cases could achieve initial remissions with intensive chemotherapy, although relapse was common and durable molecular clearance of BRAF was rare [17,18]. Patients with MDS and CMML demonstrated relatively more favorable outcomes, with median OS of approximately 16–22 months in larger cohorts [11,14,17]. Notably, Zhang et al. (2014) reported improved survival among CMML-1 patients with low-risk cytogenetics, a finding that was not consistently reproduced in subsequent datasets [16]. Data on MPN were limited; however, co-mutation with RAS-pathway genes was associated with aggressive disease biology and progression to AML despite therapy [20,22].

### 2.5. Published Case Reports of BRAF-Altered Myeloid Neoplasms

Six detailed case reports published between 2015 and 2025 were also identified, originating from Australia, the United States, France, and India. Patient ages ranged from early childhood to 78 years, with equal sex distribution (Table S1). Underlying diagnoses included chronic myeloid leukemia (CML), therapy-related AML, CMML, therapy-related MDS, and post–acute lymphoblastic leukemia AML. There was also one case of CMML with Langerhans cell histiocytoma, the mutation was harbored in both myeloid neoplasms. BRAF p.V600E was present in five of six cases, while the remaining report described a BRAF-wild-type patient with MAPK pathway activation. Co-mutations included KRAS, TET2, and SRSF2, often accompanied by high-risk cytogenetics such as t(9;11) KMT2A–MLLT3, t(9;22) BCR–ABL1, +8, del(7q), and +5. Treatments ranged from conventional chemotherapy and hypomethylating agents to MAPK inhibitors, with responses varying from transient cytoreduction to complete remission. Durable responses were rare; relapses often coincided with KRAS-mutated subclones. Death occurred in four cases, typically within a short time frame due either to disease progression or treatment-related complications. One patient was reported to be in preparation for haploidentical bone marrow transplantation at last follow-up.

## 3. Discussion

BRAF mutations are rare in myeloid neoplasms, occurring in <1% of unselected cohorts and more commonly in CMML and therapy-related AML [10,11,14,15,16,17,18,19,20,21,22]. Most reported BRAF mutations were clustered within the kinase domain, specifically involving the activation segment (exon 15, including V600). Despite the overall poor outcomes, achieving clearance of BRAF mutations was associated with longer survival, and this clearance may be augmented by HSCT. These findings highlight the clinical relevance of identifying BRAF mutations despite their low prevalence.

When compared with other hematologic malignancies, the role of BRAF in myeloid disease is far less definitive. In hairy cell leukemia, BRAF V600E is disease-defining and therapeutically targetable [8,9]. In Langerhans cell histiocytosis and Erdheim–Chester disease, BRAF or MAPK pathway mutations occur in 50–70% of cases and underpin the dramatic success of BRAF/MEK inhibitors, now approved in these settings [30,31,32,33,34]. In multiple myeloma, BRAF V600E is rare but associated with more aggressive disease and inferior outcomes [35,36]. By contrast, in myeloid neoplasms, BRAF mutations arise infrequently and within a heterogeneous genomic background, limiting their utility as stand-alone biomarkers or therapeutic targets [10,11,14,15,16,17,18,19,20,21,22]. Indeed, some studies such as Shin et al. (2016) have reported no BRAF mutations at all, underscoring the variability across cohorts [37].

The biological implications of these findings are important. Canonical class 1 mutations, such as V600E, act as constitutively active monomers, while class 2 and class 3 variants rely on dimerization or upstream RAS activation. Their frequent co-occurrence with epigenetic regulators (ASXL1, TET2, DNMT3A), splicing factors (SRSF2), and RAS pathway mutations (KRAS/NRAS) suggest that BRAF is rarely an initiating lesion in myeloid disease but rather contributes to clonal evolution and progression. These co-occurrences likely reflect shared clonal architecture and disease biology rather than a direct functional interaction between BRAF and specific co-mutated genes. Given the reported adverse prognostic impact of many of these co-mutations, the available data do not allow reliable assessment of an independent biological or prognostic contribution attributable specifically to BRAF mutations. However, we also acknowledge that some cases describe BRAF as the sole detectable abnormality (within the limits of the assays used), raising the possibility that BRAF could act as a more central driver in select patients. Because the included studies span different testing eras and platforms (PCR vs broader NGS), improvements in sequencing breadth and sensitivity may increasingly clarify whether there exists a subset with more “isolated” BRAF lesions or more interpretable clonal architecture.

Reports of clonal dynamics support this view: in some patients, BRAF persists or expands at relapse, while in others it is replaced by alternative drivers, underscoring its instability as a therapeutic target [11,14].

From a clinical standpoint, BRAF testing may be most relevant in contexts where it is enriched, such as CMML and therapy-related AML [11,14,19]. The relative enrichment of BRAF mutations in myeloid neoplasms with prominent monocytic differentiation, including CMML and acute monocytic leukemia, suggests a potential lineage-specific biological context. Although MAPK signaling is ubiquitous across cell types, monocytic lineage cells exhibit heightened dependence on tightly regulated MAPK activity during differentiation and inflammatory activation [38]. Parallels can be drawn with histiocytic disorders such as Langerhans cell histiocytosis and Erdheim–Chester disease, which arise from monocyte-derived cells and are characterized by frequent MAPK pathway mutations. These observations raise the possibility that monocytic-lineage cells may be particularly permissive to MAPK-driven clonal expansion; however, this association remains speculative and requires dedicated mechanistic and functional studies for validation. Standard therapies remain ineffective, with short survival in AML and modest benefit in MDS and CMML. Responses to BRAF/MEK inhibitors have been observed but are typically transient, without durable molecular clearance [11,14,30,31,32,33,34]. Achieving molecular mutation clearance has been reported to be prognostically meaningful, and HSCT may offer a survival benefit when clearance is attained. However, this observation is currently supported primarily by a single retrospective study (Tariq et al.) and therefore remains hypothesis-generating and in need of validation in larger, independent cohorts and prospective studies [11,14]. These observations suggest that BRAF clearance could be explored as a potential biomarker of treatment response.

Several limitations affect interpretation. Most data come from retrospective cohorts or case reports with a small number of BRAF-positive cases. Testing strategies varied; while earlier studies used PCR-based hotspot assays, more recent cohorts used NGS, complicating prevalence estimates [10,11,12,13,14,15,16,17,18,19,20,21,22,39]. PCR-based approaches are inherently limited in their ability to detect non-hot-spot substitutions, indels, and low–allele frequency variants, potentially leading to underestimation of both the true prevalence and the full mutational spectrum of BRAF alterations in earlier cohorts. Treatment approaches were heterogeneous and often anecdotal, and publication bias may favor reports of positive targeted therapy outcomes. Although publicly available genomic databases may provide additional insights into mutation frequency and co-mutation patterns, the rarity of BRAF alterations in myeloid neoplasms, coupled with variable diagnostic annotation and clinical granularity, limits their utility for definitive genotype–phenotype correlations in this setting.

Future work should prioritize prospective multicenter studies with standardized sequencing to define prevalence and outcomes more precisely. Functional studies are needed to clarify the significance of non-V600E variants, particularly class 2 and 3 mutations. Clinical trials of BRAF and MEK inhibitors, ideally in rational combinations with chemotherapy, HMAs, or HSCT, will be required to establish whether targeting MAPK signaling can provide a durable benefit. Finally, systematic evaluation of molecular clearance as a biomarker should be incorporated into trial design.

## 4. Conclusions

BRAF mutations in myeloid neoplasms are rare but clinically relevant. They are enriched in CMML and therapy-related AML, usually occur in a complex genomic background, and portend poor outcomes. Unlike HCL or histiocytic disorders, where BRAF is a defining driver, in myeloid neoplasms, these mutations appear secondary and unstable. While targeted therapy responses have been modest and short-lived, clearance of BRAF mutations and subsequent HSCT may provide a path to improved outcomes. Collaborative efforts will be essential to clarify the prognostic and therapeutic role of this rare subset.

## Figures and Tables

**Table 4:** Clinicogenetic features, treatment patterns, and outcomes of BRAF-mutated myeloid neoplasms by disease subtype.

Feature	AML	MDS	MPN	CMML
Cytogenetics	Adverse cytogenetics common: KMT2A rearrangements, complex karyotypes, monosomy 7, +8	ASXL1, TET2, SRSF2; occasional monosomy 7 or complex karyotype; sometimes normal	Usually unremarkable; small sample size; can occur with or without JAK2, CALR	Often diploid but some intermediate/high risk; histiocytic overlap noted
Co-Mutations	RAS-pathway genes (KRAS, NRAS), TP53, DNMT3A, ASXL1; secondary AML shows heavier burden	ASXL1, TET2, SRSF2 most frequent	Canonical drivers (JAK2, CALR) may be present	TET2, SRSF2, ASXL1, RAS-pathway mutations
Treatment Patterns	Intensive induction chemotherapy; MAPK inhibitors in relapsed/refractory; transient remissions	Hypomethylating agents; rare molecular clearance; modest survival gain; no large MAPK data	Conventional therapy (hydroxyurea, ruxolitinib); no consistent BRAF-targeted use	Hydroxyurea, hypomethylating agents; MAPK inhibitors for overlap/refractory; short responses
Outcomes	Median OS ~4–7 months; poor responses; relapse with RAS-pathway persistence	Median OS 16–22 months; better outcomes in diploid cytogenetics, low co-mutation burden	Limited data: indolent/moderate unless transformation occurs	Median OS 18–24 months; some transform to AML or aggressive histiocytic disease

## Data Availability

This review is based on previously published studies. No new datasets were generated or analyzed during the current study.

## Supplementary Materials

The following supporting information can be downloaded at: https://www.mdpi.com/article/10.3390/biomedicines14030672/s1. Table S1: Clinical and Molecular Characteristics of BRAF-Mutated Myeloid Neoplasm Case Reports. Table S2: Clinical outcomes of BRAF-mutated myeloid neoplasm cases.

## Abbreviations

The following abbreviations are used in this manuscript:

AML	Acute Myeloid Leukemia
APL	Acute Promyelocytic Leukemia
BRAF	B-Raf Proto-Oncogene, Serine/Threonine Kinase
CMML	Chronic Myelomonocytic Leukemia
HCL	Hairy Cell Leukemia
HSCT	Hematopoietic Stem Cell Transplantation
MAPK	Mitogen-Activated Protein Kinase
MDS	Myelodysplastic Syndromes
MPN	Myeloproliferative Neoplasms
