# Supplementary material for: BRAF Mutations in Myeloid Neoplasms: Prevalence, Co-Mutation Landscape, and Clinical Outcomes—A Comprehensive Review
Source: Biomedicines. 2026 Mar 15;14(3):672. doi: 10.3390/biomedicines14030672 (PMC13024105; doi:10.3390/biomedicines14030672)
Supplement: Supplementary file 1 [file biomedicines-14-00672-s001.zip › biomedicines-4072415-supplementary.pdf]

Supplementary material:

Table S1: Clinical and Molecular Characteristics of BRAF-Mutated Myeloid Neoplasm Case Reports

Variables Studied / Author	Andrews MC et al.	McNew BR et al.	Konstantinou et al.	Tedjaseputra et al.	Mamidala et al.	Wander et al.
Year	2015	2017	2020	2019	2025	2017
Country	Australia	United States	France	Australia	India	United States
Age	26	13	78	75	NR (toddler)	44
Gender	Male	Female	Female	Male	Male	Female
Underlying Disease	CML, chronic phase (BCR–ABL1+)	Therapy-related AML, M4–M5	Multiorgan LCH with CMML-1	CMML-transformed AML-M5b; EMD	MN-pCT → AML-M5 (post B-ALL)	t-MDS (RAEB-2) → AML
BRAF Variant	Wild-type (no V600E)	p.V600E (VAF 5.4%)	p.V600E (VAF NR)	p.V600E (VAF NR)	p.V600E (VAF NR)	p.V600E (VAF 43.3% at AML; 14.5%→1.7% on therapy; 38.8% at relapse)
Co-Mutations	NR	KRAS Q61R (36%), KRAS G12D (2.9%)	NR	TET2 (pathogenic), SRSF2 (pathogenic)	NR	KRAS Q61H (8%), G13D (7%), G12D (5%), G12A (2%) — all at relapse
Cytogenetics	t(9;22); BCR–ABL1	t(9;11); KMT2A–MLLT3 (MLL-AF9)	NR	+8; del(7q); +5	t(9;11); KMT2A–MLLT3 (MLL-AF9)	t(9;11); KMT2A–MLLT3 (MLL-AF9)
Treatment	Dabrafenib + Trametinib (for melanoma)	Leukapheresis; low- then high-dose Ara-C (no MAPK inhibitor)	Vemurafenib (960 mg BID → 720 mg BID)	Prednisolone + hydroxyurea; azacitidine 75 mg/m² d1–7 q28d (no MAPK inhibitor)	7+3 (Ara-C + daunorubicin)	Dabrafenib + Trametinib (prior: decitabine;

						Ara-C+topotecan, refractory)
Response	Complete hematologic response (week 35); no molecular/cytogenetic remission	Cytoreduction only; no remission	LCH CR at 6 mo; CMML partial HI	Rapid HI; BM blasts 1.5%	Morphologic CR; FCM-MRD <0.1%; RT-PCR MRD for t(9;11) positive	Circulating blasts cleared; BM blasts 35%→9%
Outcome	Counts controlled to week 48; therapy stopped for melanoma progression	Death on hospital day 6–7 (hyperleukocytosis complications)	Relapse ~24 mo; death 4 mo after relapse	Progressive EMD → DIC/renal failure; death ~3 mo	Planned haploidentical BMT	Relapse with KRAS clones; death ~4 mo after diagnosis

Abbreviations: AML, acute myeloid leukemia; ALL, acute lymphoblastic leukemia; Ara-C, cytarabine; BCR–ABL1, breakpoint cluster region–Abelson 1; BM, bone marrow; BMT, bone marrow transplant; CMML, chronic myelomonocytic leukemia; CR, complete remission; DIC, disseminated intravascular coagulation; EMD, extramedullary disease; FAB, French–American–British; FCM, flow cytometry; HI, hematologic improvement; KMT2A-r, KMT2A rearrangement; LCH, Langerhans cell histiocytosis; MAPK, mitogen-activated protein kinase; MN-pCT, myeloid neoplasm post-cytotoxic therapy; MRD, measurable residual disease; NR, not reported; q28d, every 28 days; RAEB-2, refractory anemia with excess blasts-2; RT-PCR, reverse transcription PCR; VAF, variant allele frequency; WT, wild-type.

Table S2:. Clinical outcomes of BRAF-mutated myeloid neoplasm cases.

ID	Study	Survival	Follow-up	Other notes
1	Abuasab et al. (2024)	AML OS median 23 mo; improved with clearance/HSCT; CMML 16 mo; MDS 22 mo; MF not reached	Median follow-up 68 mo	Most mutations at diagnosis; some acquired at progression
2	Zhang et al. (2014)	Improved OS (HR=-2.53, p=0.04)	NA	2 AML transformations
3	Christiansen et al. (2005)	Median OS 1 month	NA	Patients received topoisomerase II inhibitors, alkylating agents, RT; secondary cancers
4	Fei et al. (2024)	AML: poor prognosis; median OS 126 days	5/14 alive at last follow-up	BRAF mutation stable/lost at relapse; some response to BRAF/MEK inhibitors
5	George et al. (2024)	Both patients died within days of diagnosis	NA	—
6	Abu-Shihab et al. (2023)	Median OS 7 mo; CR/CRi 39%; no VAF impact	31/35 deceased at last follow-up	Responses similar across therapy types; relapse: BRAF stable or lost with IQGAP3 acquired
7	Kandarpa et al. (2017)	Improved response, tolerated therapy	NA	—
8	Santos et al. (2014)	Worse OS with NRAS/BRAF (HR=11.57, p=0.001); effect not independent in multivariable	NA	Transformed to AML; CR with induction; allo-HCT; relapse at day +35
9	Papaemmanuil et al. (2016)	Worse prognosis, HR=1.4 (p=0.009)	Median FU 5.9 y	Treatment: ICE ± ATRA; allo-HCT in high-risk
10	Xu et al. (2017)	3/4 died ≤12 mo; 1 in remission	NA	2 with extramedullary involvement
11	Lee et al. (2004)	NA	NA	NA
12	Lee et al. (2025)	Median OS was 5.67 months (range, 0.13-90.7 months). VAF (≥15%) has shorter median OS (P = 0.132)	NA	NA

Abbreviations: OS = overall survival; CR = complete remission; CRi = complete remission with incomplete hematologic recovery; HSCT = hematopoietic stem cell transplantation; FU = follow-up; ICE = idarubicin, cytarabine, etoposide; ATRA = all-trans retinoic acid; VEN = venetoclax; RT = radiotherapy.
